# Real-Time Fast Scan Cyclic Voltammetry Detection and Quantification of Exogenously Administered Melatonin in Mice Brain

**DOI:** 10.3389/fbioe.2020.602216

**Published:** 2020-11-24

**Authors:** Elisa Castagnola, Elaine M. Robbins, Kevin M. Woeppel, Moriah McGuier, Asiyeh Golabchi, I. Mitch Taylor, Adrian C. Michael, Xinyan Tracy Cui

**Affiliations:** ^1^Department of Bioengineering, University of Pittsburgh, Pittsburgh, PA, United States; ^2^Department of Chemistry, University of Pittsburgh, Pittsburgh, PA, United States; ^3^Center for Neural Basis of Cognition, University of Pittsburgh, Pittsburgh, PA, United States; ^4^Department of Chemistry, Saint Vincent College, Latrobe, PA, United States; ^5^McGowan Institute for Regenerative Medicine, University of Pittsburgh, Pittsburgh, PA, United States

**Keywords:** fast scan cyclic voltammetry, melatonin, fouling, carbon fiber microelectrodes, brain, electrochemical impedance spectroscopy

## Abstract

Melatonin (MT) has been recently considered an excellent candidate for the treatment of sleep disorders, neural injuries, and neurological diseases. To better investigate the actions of MT in various brain functions, real-time detection of MT concentrations in specific brain regions is much desired. Previously, we have demonstrated detection of exogenously administered MT in anesthetized mouse brain using square wave voltammetry (SWV). Here, for the first time, we show successful detection of exogenous MT in the brain using fast scan cyclic voltammetry (FSCV) on electrochemically pre-activated carbon fiber microelectrodes (CFEs). *In vitro* evaluation showed the highest sensitivity (28.1 nA/μM) and lowest detection limit (20.2 ± 4.8 nM) ever reported for MT detection at carbon surface. Additionally, an extensive CFE stability and fouling assessment demonstrated that a prolonged CFE pre-conditioning stabilizes the background, *in vitro* and *in vivo*, and provides consistent CFE sensitivity over time even in the presence of a high MT concentration. Finally, the stable *in vivo* background, with minimized CFE fouling, allows us to achieve a drift-free FSCV detection of exogenous administered MT in mouse brain over a period of 3 min, which is significantly longer than the duration limit (usually < 90 s) for traditional *in vivo* FSCV acquisition. The MT concentration and dynamics measured by FSCV are in good agreement with SWV, while microdialysis further validated the concentration range. These results demonstrated reliable MT detection using FSCV that has the potential to monitor MT in the brain over long periods of time.

## Introduction

Melatonin (MT), the pineal gland’s major secretory product under dark conditions in all mammals, including humans (Farías et al., 2012), is well known for its role in circadian rhythms and modulation of the immune system (Maestroni et al., 1986; Cagnacci et al., 1992; Szczepanik, 2007; Salehi et al., 2019). MT is already recognized for the treatment of sleep disorder (Jan et al., 1994; Buscemi et al., 2004; Xie et al., 2017). Furthermore, MT is a multitasking molecule (Reiter et al., 2010) with anti-inflammatory, antioxidant (Reiter et al., 2003; Hardeland, 2005; Reiter et al., 2016), neuroprotective (Túnez et al., 2004; Escribano et al., 2014), anti-nociceptive (Mukherjee et al., 2010; Wilhelmsen et al., 2011), and anticonvulsant properties (Fauteck et al., 1999; Banach et al., 2011; Aydin et al., 2015). As an antioxidant, MT prevents neurotoxicity, oxidative stress, and neuroinflammation in experimental models of Parkinson’s (Antolín et al., 2002; Mayo et al., 2005) and Alzheimer’s disease (Dragicevic et al., 2011; Rosales-Corral et al., 2012; Hossain et al., 2019; Luengo et al., 2019). In addition, MT’s neuroprotective functions have shown therapeutic potentials for the treatment of amyotrophic lateral sclerosis (Zhang et al., 2013), Huntington’s disease (Túnez et al., 2004; Escribano et al., 2014), and cerebral ischemia (Borlongan et al., 2000; Cuzzocrea et al., 2000), while its anti-inflammatory and anti-nociceptive actions can alleviate chronic pain both in experimental (Mukherjee et al., 2010; Wilhelmsen et al., 2011) and clinical (Korszun et al., 1999; Citera et al., 2000) studies. Due to its lipophilic character, MT can easily cross the blood–brain barrier and enter both glia and neurons, making it an excellent therapeutic choice for central nervous system (CNS) disorders (Miller et al., 2015; Golabchi et al., 2018; Salehi et al., 2019). MT’s numerous physiological roles and potential therapeutic actions justify a strong need for real-time detection and monitoring of MT in the brain of laboratory animal models.

Previously, we have demonstrated detection of exogenously administered MT in anesthetized mouse brain using square wave voltammetry (SWV) (Castagnola et al., 2020). Fast scan cyclic voltammetry (FSCV) is an electroanalytical technique with higher temporal resolution (usually 100 ms) (Robinson et al., 2003; Swamy and Venton, 2007; Wood and Hashemi, 2013; Oh et al., 2016; Ou et al., 2019; Puthongkham and Venton, 2020) and, when used in combination with carbon fiber electrodes (CFEs), can achieve detection of sub-second fluctuations in neurotransmitter concentrations in real-time in the brain (Keithley et al., 2011; Wood and Hashemi, 2013; Nguyen and Venton, 2014; Mark DeWaele et al., 2017; Castagnola et al., 2018; Puthongkham and Venton, 2020). However, being primarily a background subtraction technique, FSCV measurements are limited to short time intervals (<90 s) due to the instability of the background currents, i.e., background drifting (Oh et al., 2016; Oh et al., 2016; Mark DeWaele et al., 2017; Meunier et al., 2019). This background drift can be attributed to a number of factors, comprising the changes occurring at the carbon surface itself—i.e., chemical reaction of electrode material, non-specific absorption of proteins, deposition of byproducts of electrochemical reactions (Harreither et al., 2016; Hensley et al., 2018; Puthongkham and Venton, 2020)—and changes in the surrounding chemical and biological neuro-environment—i.e., pH and local blood flow fluctuations (Mark DeWaele et al., 2017; Roberts and Sombers, 2018; Meunier et al., 2019). To predict the noise and extract the signal contribution from the drifting background, signal filtering (Mark DeWaele et al., 2017; Puthongkham and Venton, 2020), multivariate analyses (Hermans et al., 2008; Meunier et al., 2019), and waveform manipulations combined with mathematical techniques (Oh et al., 2016; Meunier et al., 2019) have been investigated, but they require training sets (Johnson et al., 2016; Puthongkham and Venton, 2020) and/or data preprocessing (Puthongkham and Venton, 2020). On the other hand, different electrochemical procedures have been shown to restore the sensitivity of the carbon surface and preserve their current stability and sensitivity both *in vitro* and *in vivo* (Hafizi et al., 1990; Heien et al., 2003; Takmakov et al., 2010), suggesting a potential alternative to stabilize the FSCV current background. Additionally, different FSCV waveforms (Jackson et al., 1995; Ganesana et al., 2017; Hensley et al., 2018; Roberts and Sombers, 2018) have been investigated for different analytes, to identify the best holding/switching potentials and scan rate combination that maximizes sensitivity while minimize fouling (Heien et al., 2003; Cooper and Venton, 2009; Pyakurel et al., 2016; Cryan and Ross, 2019; Puthongkham and Venton, 2020). Specifically for MT, Ross’ group (Hensley et al., 2018) has developed an optimized waveform that can minimize the electrode fouling due to MT oxidation byproduct deposition. Using this waveform, they demonstrated FSCV detection of MT in lymph node slices (Hensley et al., 2018).

To the best of our knowledge, real-time FSCV detection in the brain of living animals has not been previously reported. Here, we report a prolonged CFE electrochemical pre-conditioning protocol that stabilized the FSCV background for multiple minutes. Using the Ross’ waveform at pre-electrochemically conditioned CFEs, we successfully demonstrated a *drift-free* FSCV detection of exogenous MT *in vivo* for 3 min. To demonstrate and support the reliability of this FSCV approach, we (1) performed an extensive evaluation of CFE stability and fouling in the presence of 5 μM MT *in vitro*, (2) characterized *in vivo* FSCV signal baseline, and (3) evaluated the effects of biological tissue exposure on the impedance, capacitance, and sensitivity of the CFEs. Finally, we compared the *in vivo* MT concentrations measured by FSCV with those measured by SWV in our previous study (Castagnola et al., 2020) and used microdialysis from the tissue near the CFEs for further validation.

## Materials and Methods

### Microelectrode Fabrication

#### CFE Fabrication and Pre-conditioning

Carbon fiber electrodes were fabricated as previously described in Taylor et al. (2012, 2015), and Castagnola et al. (2020). Briefly, borosilicate capillaries (0.4 mm ID, 0.6 mm OD; A-M systems Inc., Sequim, WA, United States), each containing a single carbon fiber (7 μm diameter, T650; Cytec Carbon Fibers LLC., Piedmont, SC, United States), were pulled to a fine tip using a vertical puller (Narishige, Los Angeles, CA, United States). The tip was sealed with epoxy (Spurr Epoxy; Polysciences Inc., Warrington, PA, United States) and the exposed fiber was cut 400 μm from the tip using a scalpel under an optical microscope (Szx12, Olympus). A mercury drop was injected into the barrel of the glass to create an electrical contact between the carbon fiber and a hookup wire (Nichrome; Goodfellow, Oakdale, PA, United States). CFEs were soaked in isopropyl alcohol (Bath et al., 2000) (Fisher Chemical, United States) for 20 min prior to use. The entire procedure that involved handling mercury was performed under a chemical fume hood, with the user wearing gloves, eye protection, a lab coat, and mask. After use, all electrodes and other mercury-contaminated materials were disposed of according to protocols required by Environmental Health and Safety of the University of Pittsburgh, in compliance with U.S. Department of Labor Occupational Safety and Health Administration compliance guideline 1910.120.

Carbon fiber electrode surfaces were preconditioned by applying the Ross’ FSCV waveform (0.2–1.3 V versus Ag/AgCl, 600 V/s) at 10 Hz overnight prior to the beginning of each experiment.

#### Microdialysis Probes and Procedures

Concentric style microdialysis probes were fabricated as previously described (Nesbitt et al., 2015; Varner et al., 2016; Robbins et al., 2019). Briefly, the probes were constructed in-house using hollow fiber membranes (18 kDa molecular weight cutoff, Spectra/Por RC, Spectrum, Rancho Dominguez, CA, United States) with a 280 μm OD, cut to a length of 1.5 mm. The inlet and outlet lines were made from fused silica capillary (75 μm ID, 150 μm OD, Polymicro Technologies, Phoenix, AZ, United States). Probes were perfused with artificial cerebrospinal fluid (aCSF; 142 mM NaCl, 1.2 mM CaCl_2_, 2.7 mM KCl, 1.0 mM MgCl_2_, 2.0 mM NaH_2_PO_4_, pH 7.4) at 2 μL/min using a gastight syringe (1000 Series Gastight Hamilton^TM^, Reno, NV, United States) driven by a syringe pump [Harvard Apparatus Model 11 (55-1199), Holliston, MA, United States] starting prior to insertion into the brain and their functionality was verified by placing the probe membrane into a beaker of 5 μM MT and detecting MT at the outlet. As aCSF does not have a carbonate buffer system, the pH was adjusted to pH 7.4, using NaOH and HCl (Sigma–Aldrich, MO, United States). Dialysate was analyzed for MT on a Shimadzu LCMS-2020 liquid chromatography mass spectrometer (LC-MS) using atmospheric pressure chemical ionization (APCI) by monitoring the [M + H]^+^ peak at 233.1 *m/z*. MT was separated using a gradient of 10–90% solvent A to solvent B over 10 min, where solvent A was acetonitrile with 0.1% formic acid, and solvent B was water with 0.1% formic acid. Data were analyzed using Shimadzu Lab Solution software and in MATLAB (Mathworks). Dialysate was diluted with ultrapure water (Nanopure, Barnstead, Dubuque, IA, United States) before analysis to protect the instrument from damage due to the high salt content of the sample.

### *In vitro* FSCV Calibration

**FIGURE 1:**
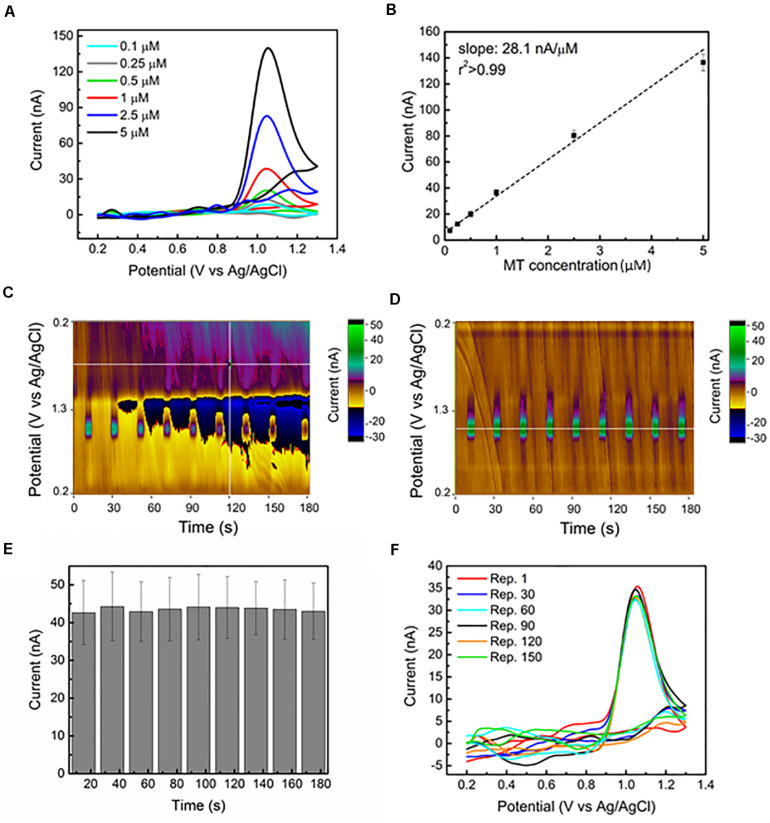
Sensitive and stable MT detection via FSCV at CFEs in aCSF: **(A)** Representative background subtracted CVs, for 0.1, 0.25, 0.5, 1, 2.5, and 5 μM bolus of MT, respectively, reveal a clear MT oxidation peak at 1.03 V. **(B)** *In vitro* FSCV MT calibration curve. The average (*n* = 6) sensitivity (background subtracted peak current versus MT concentration) is linearly correlated. **(C)** Representative color plot for nine repetitions of 1 μM MT bolus injections during a 3-min FSCV collection performed using a 15 min pre-conditioned CFE shows an unstable background, starting from 30 s. The changes in current (at the 1.03 V oxidation peak) following the injection of 1 μM bolus of MT every 15 s are not clearly detectable because of the background drift. The electrochemical drift increased with time and after about 2 min, the current changes due to MT injections are no longer discernible. **(D)** Representative color plot for nine repetitions of 1 μM MT bolus injections during a 3 min FSCV collection performed using an overnight preconditioned CFE show a stable background. The changes in current (at the 1.03 V oxidation peak) following the injection of 1 μM bolus of MT every 15 s are clearly detected along the 3-min window. **(E)** Bar plot reporting the background-subtracted current amplitudes (average ± SEM, *n* = 6 CFEs) of the oxidation peak at 1.03 V collected for the nine repetitions along the 3 min from the overnight conditioned CFEs. The average current peak amplitudes (first: 42.7 ± 8.5 nA, last 43.0 ± 7.5 nA, *n* = 5) are not significantly affected by time during the nine different injections [repeated measures ANOVA, *F*(7,42) = 0.235, *p* > 0.05]. **(F)** Background subtracted CV plots at different time points (from Repetition 1, corresponding to time zero, to Repetition 150, corresponding to 8 h of FSCV scanning) in response to 1 μM bolus injections of MT every 3 min for more than 8 h. The reported plots are almost identical along this time, with a 0.74% variation in peak current amplitude.

**FIGURE 2:**
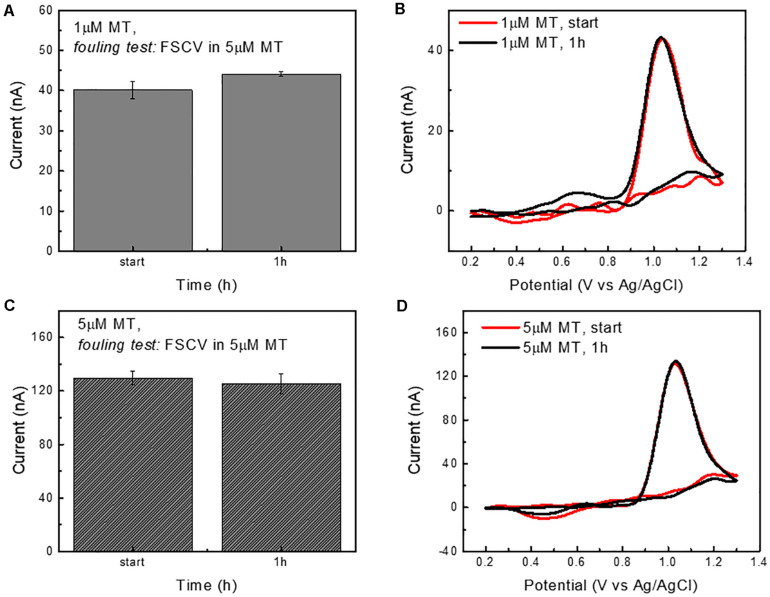
CFE fouling test in the presence of MT (continuous FSCV scanning). **(A)** Bar plot reporting the current amplitudes (average ± std, *n* = 5 repetitions) of the oxidation peaks collected in response to 1 μM bolus injections of MT at time zero and after 1 h of CFE FSCV continuous scanning in 5 μM MT concentration in aCSF. The average current peak amplitudes (40.1 ± 3.7 and 44.1 ± 1.0 nA) are not significantly different. **(B)** Representative background subtracted CVs in response to 1 μM bolus injections of MT at the time 0 and after 1 h of CFE FSCV continuous scanning in 5 μM MT concentration in aCSF. **(C)** Bar plot reporting the current amplitudes (average ± std, *n* = 5 repetitions) of the oxidation peak collected in response to 5 μM bolus injections of MT at time zero and after 1 h of CFE FSCV continuous scanning in 5 μM MT concentration in aCSF. The average current peak amplitudes (first: 130 ± 10 and 125.3 ± 7.5 nA after 1 h) are not significantly different. **(D)** Representative background subtracted CVs in response to 5 μM bolus injections of MT at the time 0 and after 1 h of FSCV.

Fast scan cyclic voltammetry was performed with an EI 400 potentiostat (Ensman Instruments; Bloomington, IN, United States) and the CV Tar Heels LabVIEW program (CV Tar Heels v4.3, University of North Carolina at Chapel Hill, Chapel Hill, NC, United States). The headstage gain was set to 10^6^ V/A. Data were analyzed using HDCV software (UNC Chapel Hill). The electrode was scanned using the Ross waveform, corresponding to a triangular waveform with a positive holding potential of 0.2 V, a 1.3 V switching potential, applied using a 600 V/s scan rate at 10 Hz (Hensley et al., 2018). MT was identified by inspection of the background-subtracted cyclic voltammograms. The MT oxidation peak occurred at 1.03 V (Figures 1A,F, 2B,D, 4C). Electrodes were calibrated using 0.1–5 μM MT concentrations dissolved in aCSF. The different concentrations were diluted starting from a freshly prepared 1 mM MT solutions. Calibration was performed in flow cell equipped with a pneumatically actuated injection valve with 500 μL sample loop (VICI AG International, Switzerland): flow through the system at 60 mL/h was driven with a syringe pump (Taylor et al., 2017).

Other analytes, such as DA and 5-HT that might interfere with MT detection, were tested using the same flow cell apparatus (Supplementary Section 1 and Supplementary Figures 1, 2 for more consideration about possible neurotransmitter/MT metabolite interferences).

### Electrochemical Characterization

Electrochemical impedance spectroscopy (EIS) measurements were used to investigate the electrode/solution interface prior to, during, and following implantation in the brain tissue, as previously described (Taylor et al., 2019; Castagnola et al., 2020). During the EIS measurements, a sine wave (10 mV RMS amplitude) was superimposed onto the open circuit potential while varying the frequency from 1 to 10^5^ Hz. EIS was carried out using a potentiostat/galvanostat (Autolab, Metrohm, United States). The *in vitro* EIS was performed in aCSF in a three-electrode electrochemical cell set-up with a platinum counter electrode and an Ag/AgCl reference electrode. *In vivo*, a screw was used as counter electrode and the Ag/AgCl reference electrode was electrically connected via a salt bridge to the surface of the brain. Specifically, ca. 1 × 1 cm^2^ square of Kimwipe (Kimtech^TM^ Science Brand) was rolled and pulled through a pipet tip (volume 100–1250 μL, SureOne, Fisher Scientific, MA). The pipet tip was filled with aCSF and the reference electrode (CHI111P, CH Instruments Inc., TX) inserted into the pipet tip. The wet exposed part of the kimwipe is place in contact with the brain through a small pinhole craniotomy. Equivalent circuit modeling was performed using EIS Spectrum Analyzer 1.0. Model optimization was performed using a modified Levenburg–Marquardt algorithm with parametric weighting. A chi-square goodness of fit test was used to assess equivalent circuits describing the data. The variation of the FSCV current, defined here as the total current resulting from all faradaic and non-faradaic processes (i.e., background current) during the triangle voltage application, was recorded, *in vitro* and *in vivo*, using the EI 400 potentiostat and the CV Tar Heels LabVIEW program, and was evaluated by calculating (1) the charge storage capacity (CSC, mC/cm^2^) as $C\,S\,C=\frac{\left|\int i\,dt\right|}{A\,r\,e\,a\,C\,F\,E}$ in an entire CV cycle and (2) the capacitance (C,nF) as $C=\frac{\left|\int i\,dV\right|}{2*s\,c\,a\,n\,r\,a\,t\,e*p\,o\,t\,e\,n\,t\,i\,a\,l\,w\,i\,n\,d\,o\,w}$.

### *In vivo* Procedures

All procedures involving animals were approved by the Institutional Animal Care and Use Committee of the University of Pittsburgh. CFEs were acutely implanted in the visual cortex of male mice (C57BL/6J, 8–12 weeks, 22–35 g; Jackson Laboratory, Bar Harbor, ME, United States): this strain was selected for their low endogenous MT levels (Roseboom et al., 1998). Anesthesia was induced with 1.5–2% isoflurane mixed with oxygen flow at 1 L/min and maintained with 1.25–1.5%. Body temperature was maintained at 37°C with a thermostatic heating pad (Harvard Apparatus, Holliston, MA, United States) and eye lubrication was maintained with lacrigel (Dechra Puralube Vet Ointment). The implantation procedures have been performed as previously reported (Castagnola et al., 2020). Briefly, the animal’s head was fixed in a stereotaxic frame (Narishige International USA, Inc.). In *n* = 12 mice, a pinhole craniotomy was made over the visual cortex (1.0 mm anterior to lambda and 1.5 mm lateral from midline) with a high-speed drill (0.007 drill bit, Fine Science Tools, Inc., Foster City, CA, United States). An additional pinhole craniotomy was made to establish the connection to the reference electrode via a salt bridge. CFEs were lowered 0.8 mm below the brain surface.

Following insertion into the tissue, the CFE was subjected to the Ross FSCV waveform applied at 10 Hz for at least 30 min for stabilization. After the stabilization period, the animal received an intraperitoneal (i.p.) injection of 180 mg/kg MT (*n* = 5) (99 + % Alfa Aesar, Haverhill, MA, United States) or a volume-matched injection of saline (*n* = 2), followed by 3 min of FSCV recording. Similarly to our previous studies (Golabchi et al., 2018; Castagnola et al., 2020), 10 μL dimethylsulfoxide (DMSO, 99% Alfa Aesar, Haverhill, MA, United States) was added in order to improve the solubility of the MT in 100 μL saline vehicle. 10 μL of DMSO was also added to the saline vehicle for the control group, in order to exclude the DMSO effect on the background stability.

In another *n* = 5 mice, FSCV and microdialysis were performed simultaneously. In this case, the borosilicate glass tube of a CFE was glued to the stainless-steel housing of microdialysis probe. A larger craniotomy was performed to permit insertion of the devices. Then the microdialysis probes were perfused with aCSF at 2 μL/min using a gastight syringe driven by a syringe pump (Harvard Apparatus, Holliston, MA, United States) for 1 h, while electrochemical stabilization of the CFE surface was performed. Immediately after, mice received a single 180 mg/kg, i.p. injection of MT, and FSCV measurements were performed and dialysate collected.

At the end of the *in vivo* measurement, the CFEs were removed from the brain with the help of the micromanipulator, washed with abundant DI water followed by post calibration. Then, the Ross’ waveform was applied to the CFEs overnight (≥12 h) at 600 V/s or, a more aggressive FSCV waveform (−0.5 to 1.9 V versus Ag/AgCl and back at 400 V/s) was applied for 2 min, to remove the biological encapsulation and restore the CFE surface (see Supplementary Section 3 and Supplementary Figures 4, 9, 10). In our previous study, we have demonstrated that this aggressive FSCV waveform (−0.5 to 1.9 V versus Ag/AgCl and back at 400 V/s) was able to clean the CFEs from biological matter encapsulation after the extraction from the brain, preserving the CFE integrity (Castagnola et al., 2020). The post-calibration was repeated ≥ 4 h after the electrochemical cleaning procedure.

### Statistical Analyses

Statistical analyses were conducted using IBM SPSS software (v22, IBM Corp., Armonk, NY, United States) and Origin Pro 8.1 (OriginLab Corp., Northampton, MA, United States). One-way ANOVA with Bonferroni post-tests was used to compare changes in the sensitivity of CFEs to MT in presence of MT (continuous FSCV scanning) *in vitro*. One-way repeated measures ANOVA with Bonferroni post-tests was used to calculate the variations in MT sensitivity over time during consecutive MT injections *in vitro*, and the changes in CSC (mC/cm^2^) and capacitance (C,nF), as defined in Section “Electrochemical Characterization,” over time *in vivo.* Two-way repeated measures ANOVA with Bonferroni post-tests was used to compare the EIS changes before, during, and following brain implant. Significance was determined at *p* < 0.05.

## Results and Discussion

### *In vitro* FSCV Sensitivity and Selectivity

Melatonin oxidizes at carbon-based electrodes following a three-step reaction (Xiao-Ping et al., 2002; Vasantha and Chen, 2005; Hensley et al., 2018) involving the formation of a quinoneimine, which is both highly reactive and susceptible to electropolymerization, causing undesirable adsorption products to foul the electrode surface (Xiao-Ping et al., 2002; Vasantha and Chen, 2005; Hensley et al., 2018). To minimize the extent of MT fouling, the Ross’ group optimized a FSCV waveform that combines a positive holding potential (0.2 V) and a faster scan rate (600 V/s) (Hensley et al., 2018). Furthermore, the use of a 1.3 V switching potential has shown to maximize the sensitivity of the primary oxidation peak (Hensley et al., 2018).

Using this waveform, we evaluated the sensitivity of our CFE in detecting MT *in vitro* with a range of MT concentration of 0.1–5 μM in aCSF. The background subtracted CVs corresponding to this range of concentrations demonstrate clear oxidation peaks at 1.03 V, as reported in Figure 1A. The sensitivity is determined to be 28.1 nA/μM, based on the linear regression slope of the maximum faradaic current versus MT concentration plot. The average calibration plot (±SEM, *n* = 6) follows a linear trend (Figure 1B), including linear regression fit (*r*^2^ > 0.990). Our sensitivity (28.1 nA/μM) is four times higher than what was reported in Hensley et al. (2018), which is likely due to the four times larger surface area of our CFEs. The lower detection limit (LOD), defined as three times the standard deviation of the noise, was estimated to be 20.2 ± 4.8 nM (*n* = 7), which is the lowest LOD value reported in literature using carbon-based materials and electrochemical detection methods for MT detection (Levent, 2012; Gomez et al., 2015; Apetrei and Apetrei, 2016; Neeraj Kumar, 2017). The selectivity of MT detection at CFEs using this FSCV waveform over other electroactive species, including ascorbic acid (AA), dopamine (DA), serotonin (5-HT) and their mixtures, and H_2_O_2_ has been evaluated. The results have shown that MT can be distinguished among the most common electroactive neurotransmitters in the brain (data reported in Supplementary Figures 1, 2 and Supplementary Section 1).

### *In vitro* FSCV Stability and Electrode Fouling

The stability of CFEs is important for monitoring MT variations over an extended duration of time in vivo (Mark DeWaele et al., 2017; Roberts and Sombers, 2018). As previously mentioned, one major limitation of traditional FSCV measurements is related to the instability of the background currents, i.e., background drift, that limits the FSCV data analysis to short time intervals (<90 s) (Mark DeWaele et al., 2017; Meunier et al., 2019).

Here, to evaluate the effect of a prolonged CFE pre-conditioning on the background stability and MT sensitivity over time, we applied the Ross’ FSCV waveform (0.2–1.3 V versus Ag/AgCl, 600 V/s) at 10 Hz to CFE electrodes in aCSF overnight prior to the beginning of each experiment. This corresponds to cycling the CFEs for about 2 h at 60 Hz using the same waveform, longer than what previously reported (15 min at 60 Hz) (Takmakov et al., 2010; Meunier et al., 2020). This pre-conditioning procedure stabilized the current background *in vitro*, as can be observed in the representative color plots for nine repetitions of 1 μM MT bolus injections during a 3-min collection in aCSF (Figures 1C,D). Using a 15 min conditioned CFE, the electrochemical drift started after 30 s, increased with time and, after about 2 min, the MT oxidation peak was no longer detectable because of the background instability (Figure 1C). In contrast, after the overnight pre-conditioning of the same CFE, we did not observe any background drift along the 3-min recording window (Figure 1D). The current amplitudes (average and SEM, *n* = 5 CFEs) of the oxidation peak at 1.03 V collected for the 30 repetitions along 10 min are reported in the bar plot in Figure 1E. The averaged current peak amplitudes (first: 42.7 ± 8.6 nA, last 41.3 ± 5.5 nA, *n* = 5) are not significantly affected by time during nine consecutive injections [repeated measures ANOVA, *F*(7,42) = 0.235, *p* > 0.05].

Electrochemical procedures have been used before sensing to pretreat carbon surface and activate the surface with functional groups that may improve detection (Engstrom and Strasser, 1984; Bowling et al., 1989; Cao et al., 2019), or during sensing to regenerate electrochemically active surfaces and restore the sensitivity of carbon microelectrodes affected by irreversible non-specific adsorption of biomolecules (Takmakov et al., 2010). In particular, oxidation of carbon surfaces using triangular waveforms with a switching potential higher than 1 V (i.e., 1.3 and 1.4 V versus Ag/AgCl) has been shown to preserve the current stability for DA detection both *in vitro* and *in vivo* (Hafizi et al., 1990; Heien et al., 2003; Takmakov et al., 2010) as a result of continuous carbon surface renewal (Takmakov et al., 2010). To our knowledge, the effect of a prolonged electrochemical preconditioning, in conjunction with continuous regeneration of the CFE surface, on FSCV background stability has not been reported.

The electrochemical pretreatments can have multiple effects on the electrode surface, and can (1) affect the concentration of physisorbed impurities on the carbon surface, i.e., electrode cleanliness (Bowling et al., 1989), (2) create surface oxygen-functional groups (Engstrom and Strasser, 1984; Bowling et al., 1989; Cao et al., 2019), (3) alter the carbon substrate structure due to the formation or removal of defects, in particular affecting the graphitic edge plane density (Poon et al., 1988; Bowling et al., 1989), and (4) modify the microscopic surface area (Poon et al., 1988; Bowling et al., 1989). The extent of these effects is likely to be dependent upon the number of electrochemical cycles or duration of the treatment. Here, we monitored the impedance and the FSCV current, i.e., background current during the triangle voltage application, during the electrochemical pre-conditioning. The impedance spectra and FSCV plots at different time points are reported in Supplementary Figure 3. We observed a fast decrease in impedance and corresponding increase in FSCV current during the first 45 min. After that, these trends slowed down over time, and after 8 h of FSCV cycling at 10 Hz, both impedance and FSCV current maintained the stability for the following 16 h (under continuous cycling). The starting 45-min fast trend in impedance reduction and current increase is likely due to the separation of graphite planes (Maeda et al., 1985; Bowling et al., 1989), known to be the first step of the electrochemical activation (Bowling et al., 1989), with consequent fracturing of the graphite lattice and formation of edge plane defects. The formation of functional groups during the electrochemical conditioning has shown to further facilitate this phenomenon by creating strains in the lattice which causes edge planes to form and planes to delaminate (Bowling et al., 1989). The continuous FSCV cycling at 10 Hz with the 1.3 V switching potential likely increases the amount of oxygen groups at the surface and continues to favor this “activation process” until the maximum amount of defects is formed, at which point the carbon electrode surface reaches a stable state and we no longer observe impedance or FSCV current change upon cycling. SEM images of a pristine CFE, a CFE at which the Ross’ waveform was applied overnight (≥12 h) at 600 V/s and a CFE at which a more aggressive FSCV waveform (−0.5 to 1.9 V versus Ag/AgCl and back at 400 V/s) was applied for 2 min are reported in Supplementary Figure 4. The integrity of the CFE seems to be preserved after the electrochemical pretreatments.

To test the *in vitro* CFE stability to MT detection over time, we monitored the response of 1 μM bolus injections of MT every 3 min for more than 8 h. The background subtracted CV plots at different time points (from Repetition 1, corresponding to time zero, to Repetition 150, corresponding to 8 h of FSCV scanning) are reported in Figure 1F. Despite variations in the current baseline, the MT oxidation peak was not affected over time, presenting a minimal 0.74% variation in current amplitude.

These results indicated the effectiveness of the prolonged CFE pre-conditioning on the background stabilization and the stability of the FSCV MT sensitivity over time. However, because the CFE_S_ were exposed to MT only during the bolus MT injection (approximately 5 s), these experiments only partially represent the *in vivo* MT fouling situation. Thus, to estimate the CFE fouling in the presence of MT, CFEs were cycled using the Ross waveform for 1 h in aCSF containing a 5 μM MT concentration. The results are reported in Figure 2. The CFE sensitivity to a bolus injection of 1 μM MT and 5 μM MT was tested before and after the FSCV cycling in MT and the peak amplitudes of MT oxidation were not significantly changed, both for the detection of 1 μM MT (one-way ANOVA, *p* = 0.14; Bonferroni post-tests n.s.) (Figures 2A,B) and 5 μM MT (one-way ANOVA, *p* = 0.31; Bonferroni post-tests n.s.) (Figures 2C,D). This result suggests that the optimized waveform is effective in preserving the electrode from fouling even in the presence of a high MT concentration. Additionally, the use of a 1.3 V switching potential, regenerating a fresh CFE surface by continuous etching of the carbon electrode (Takmakov et al., 2010; Keithley et al., 2011), may helped to mitigate the fouling.

We should note that we purposefully designed a highly aggressive *in vitro* fouling resistance test by challenging the CFE with high concentrations of MT. In our previous study, 5.5 μM of MT corresponded to the peak concentration detected in the mouse brain after administration of high MT dose (180 mg/kg), which only lasted for a few minutes before MT being metabolized (Castagnola et al., 2020).

### Evaluation of CFE Fouling *in vivo* and Effect of Tissue Exposure on CFE Sensitivity

**FIGURE 3:**
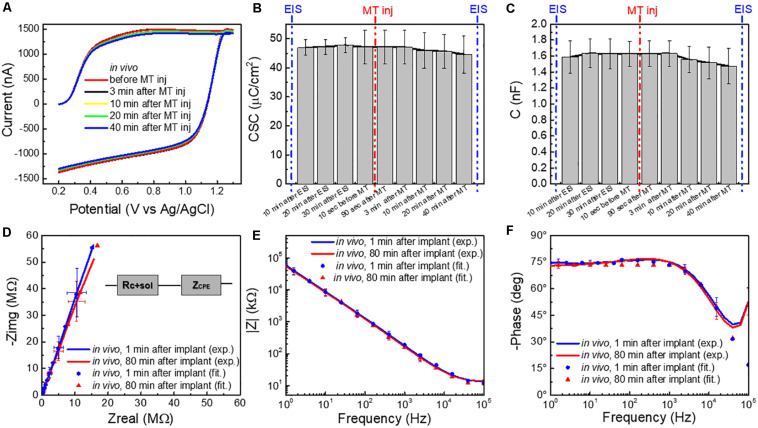
**(A)** Averaged FSCV current (for *n* = 5 CFEs) *in vivo* before and 3, 10, 20, and 40 min after MT administration. **(B)** Charge storage capacity (CSC, μC/cm^2^), and **(C)** capacitance (C,nF) (*n* = 5, average and standard deviation), calculated from the FSCV current *in vivo* at different time points: 10, 20, and 30 min after CFE implant (i.e., during *in vivo* pre-conditioning), 10 s before MT administration and 180 s, 3, 10, 20, and 40 min after MT administration (i.e., FSCV in presence of MT). **(D)** Average Nyquist plot (*n* = 6, 1 Hz–100 kHz) of CFEs measured 1 min after the implant *in vivo* (blue), at the end of the experimental session (≥80 min after implant) *in vivo* (red). The experimental data are reported in continuous lines and the corresponding curve-fitting in scattered points. The equivalent circuit model is reported in inset. **(E)** Magnitude and **(F)** phase EIS spectra (*n* = 6, 1 Hz–100 kHz) corresponding to the Nyquist in **(D)**.

Recent works from the Sombers’ group have suggested that the FSCV current can be used as a predictor of electrode fouling and sensitivity to multiple analytes *in situ* (Roberts et al., 2013; Meunier et al., 2017). Furthermore, EIS has been considered a valuable technique to probe electrode conditions and biofouling (Wilks et al., 2012; Alba et al., 2015), as well as predict electrochemical FSCV performance, using equivalent circuit models of the electrode/solution interface (Roberts et al., 2013; Meunier et al., 2020). Thus, to gain further insight about the extent of fouling *in vivo* and changes of electrode/tissue interface, we monitored (1) the EIS of the CFEs *in vivo*, upon probe implantation and after the experimental sessions (composed of a 30–40 min of pre-conditioning with FSCV, MT injection and a 40 min post-injection FSCV, total of ∼80 min) and (2) the variation of the FSCV current *in vivo* before, during and after the MT injection (Figure 3). Additionally, to estimate the effects of tissue exposure on impedance, capacitance, and sensitivity of the CFEs, (1) EIS and FSCV current were measured before implantation and after explantation in aCSF and (2) the sensitivity of CFEs before and after tissue exposure *in vitro*. All EIS data were fitted to an equivalent circuit model (Supplementary Section 2 and Supplementary Figure 5) in order to better investigate the nature of impedance changes.

**FIGURE 4:**
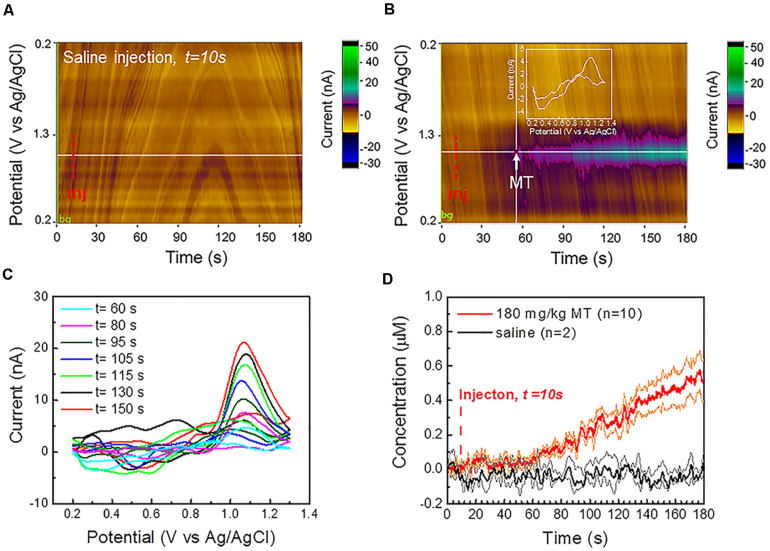
*In vivo* MT FSCV detection: **(A)** Representative color plot for a 3 min FSCV data collection after saline control injection at 10 s *in vivo* shows a stable background without observable current drift. **(B)** Representative color plot for a 3-min FSCV data collection after MT injection at 10 s (i.p., 180 mg/kg). CFEs started to detect MT about 50 s after MT administration. **(C)** Representative *in vivo* background subtracted CV at different time points following i.p. injection reveals a clear MT oxidation potential peak, that increase during the recording period. **(D)** Average *in vivo* MT concentration (±SEM) over time, detected at CFEs using FSCV before and after the i.p. administration (at 10 s) of 180 mg/kg MT (red, *n* = 10) and saline vehicle (*n* = 2, black), respectively. The orange and green lines correspond to the min and max concentration trends, calculated as mean ± SEM (i.e., mean ± standard deviation divided by the square root of the sample size) for experimental and control groups, respectively. CFEs started to detect an increase in MT concentration about 50 s following the i.p. injection and reach the concentration of 0.51 μM (0.40 μM min, 0.62 μM max) 170 s following MT administration. No change has been observed in the background-subtracted FSCV after injection of saline vehicle. 10 μL DMSO was added in order to improve the solubility of the MT in 100 μL saline vehicle, as well as in the saline vehicle for the control group, in order to exclude the DMSO effect on background stability.

*In vivo*, little change in FSCV current, during and after the MT injection was observed (Figure 3A). The CSC (mC/cm^2^) (Figure 3B) and the capacitance (C,nF) (Figure 3C), calculated from the FSCV current, did not significantly change at different time points before and after the MT injection [repeated measures ANOVA, *F*(5,25) = 0.172, *p* > 0.05 for CSC; *F*(8,32) = 0.445, *p* > 0.05, multiple comparisons Bonferroni post-tests n.s.]. Similarly, the impedance spectra (Figures 3D–F) did not significantly change during the experimental session of ≥ 80 min [two-way ANOVA with repeated measures: *F*(1,312) = 1.287, *p* = 0.2576 > 0.05].

Average Nyquist plots (*n* = 6) of the experimental data and the corresponding curve-fitting of the most appropriate equivalent circuit model, i.e., a non-ideal Randles, are reported in Figures 3D–F and Supplementary Figure 6. More details about the model and the effect of tissue exposure are provided in Supplementary Section 2. Briefly, the non-ideal Randles equivalent circuit (Figure 3D inset) consists of a solution resistance (R_sol+carbon_) in series with a constant phase element (Z_CPE_). This is consistent with results reported in the literature for CFEs glue-sealed at the tip (Meunier et al., 2020). The constant phase element (Z_CPE_) is define as: Z_CPE_ = 1/(jω)*^*n*^* Y_o_, where Y_o_ represents the double-layer capacitance and *n* is a constant related to the angle of rotation in the complex plane compared to the purely capacitive behavior (*n* = 1 for a pure capacitor) (MacDonald, 1987; Nimbalkar et al., 2018; Meunier et al., 2020). The parameters obtained by fitting the experimental data to the model are reported in Table 1.

**TABLE 1:** Equivalent circuit model fit parameters.

	R_sol+carbon_ (kΩ)	Y_o_ (nS s^n^)	*n*
*In vitro* in aCSF, pre implant	5.3	3.4	0.853
*In vivo* time 0 (immediately after insertion in the brain)	9.2	3.7	0.829
*In vivo* time ∼80 min (after conditioning and measurements)	9.0	3.8	0.815
*In vitro* in aCSF, post implant	6.9	3.6	0.833

Immediately after CFEs were inserted in the brain, we observed (1) an increase in the real impedance, with a deviation from the ideal capacitor (Supplementary Figure 6), confirmed by the decrease of *n* values obtained from the fitting (Table 1) and (2) an increase of the solution/CFE interface resistance R_sol+carbon_ (Table 1 and Supplementary Figure 7), most likely due to the more complex and resistive nature of the brain tissue compared to the aCSF, and may also be affected by the use of a different three-electrochemical system *in vivo* (Williams et al., 2007; Mercanzini et al., 2009; Meunier et al., 2020). It is important to note that the *n* values we measured from the *in vitro* EIS of CFEs are lower than what was reported in literature for similar CFEs (ca. 0.9) (Meunier et al., 2020). This is due to the effect of the overnight pre-conditioning, as the starting *n* value before pre-conditioning was 0.891. The higher Y_0_ *in vivo* is likely due to the higher ionic strength of the tissue in comparison with aCSF, which decreases the diffuse layer thickness and increases double-layer capacitance (Bard et al., 1980; Meunier et al., 2020). During the *in vivo* measurements, all circuit parameters remain relatively steady. These results showed no significant evidence of CFE fouling during the duration of the *in vivo* experiments, consistent with the *in vitro* finding.

### *In vivo* MT Detection Using FSCV

Finally, we used FSCV to detect MT in the visual cortex of mouse brains in response to an i.p. injection of 180 mg/kg MT (Albertson et al., 1981; Sugden, 1983). In our recent SWV study, we demonstrated that the i.p. administration of this dosage creates fast changes in the MT concentration in the mouse brain (Castagnola et al., 2020). Similar MT dosages have shown to possess anticonvulsant effects and to reduce the seizure rank score in rat models of epilepsy (Albertson et al., 1981; Aydin et al., 2015). MT supplements in similar concentrations have also shown to ameliorate sleep deprivation-aggravated pain behavior and neuropathic pain in rats with chronic constriction nerve injury (Huang et al., 2014). MT administered intraperitoneally has specifically demonstrated to possess a strong antinociceptive and analgesic effect (Sugden, 1983; Yu et al., 2000) and to potentiate sleep in mice and rats (Sugden, 1983; Wang et al., 2002).

Prior to the beginning of each *in vivo* experiment, CFEs were electrochemically pre-conditioned overnight in aCSF *in vitro*, as described in the previous section. After the implantation of CFEs in the visual cortex, the microelectrode surfaces were further stabilized by applying the same FSCV waveform at 10 Hz. Stable current backgrounds were observed after about 30–40 min of FSCV scanning.

The representative color plot for a 3-min FSCV background-subtracted current collection *in vivo* (Supplementary Figure 11a) and the average FSCV background (*n* = 10, black, Supplementary Figure 11b), collected before each MT recording session, show a stable background without observable current drift. Additionally, no changes were observed in the background subtracted CV after the injection of saline vehicle (Figure 4A). MT was administered i.p. at 180 mg/kg MT 10 s after the beginning of the FSCV acquisition. CFEs started to detect MT in the brain about 50 s following the i.p. injection (Figure 4B). The detection of MT using FSCV is clearly confirmed by the color plot current changes (Figure 4B) and by the background subtracted CVs, showing an increase of the current amplitude of the characteristic MT peak at 1.03 V over time, as seen in Figure 4C.

Our previous SWV studies (Taylor et al., 2019; Castagnola et al., 2020) have concluded that there is a drastic decrease in CFE sensitivity after it is explanted from the brain tissue and this is mainly attributed to the encapsulation of the CFE by biological matter pulled out during the explantation process itself. For this reason, we used the pre-calibration curve of electrode sensitivity to determine the *in vivo* MT concentrations. Measured MT concentration (±SEM) was plotted over time before and after the MT injection (*n* = 10, red, Figure 4D) and showed a linear increase after 50 s and reached 0.51 μM (0.40 μM min, 0.62 μM max) after 170 s. In comparison, the average baseline (background-subtracted FSCV) after injection of saline vehicle (*n* = 2, black, Figure 4D) remained flat.

In summary, the CFE electrochemical pre-conditioning enabled a stable FSCV background over a recording session of 3 min, more than twice the duration limit (usually < 90 s) reported in literature for FSCV measurements before the background drifting occurs, which has prevented accurate analysis of chemical fluctuations in the brain (Mark DeWaele et al., 2017; Meunier et al., 2019). This allowed us to obtain stable FSCV detection of MT *in vivo* for 3 min.

### Comparison With SWV and Microdialysis

**FIGURE 5:**
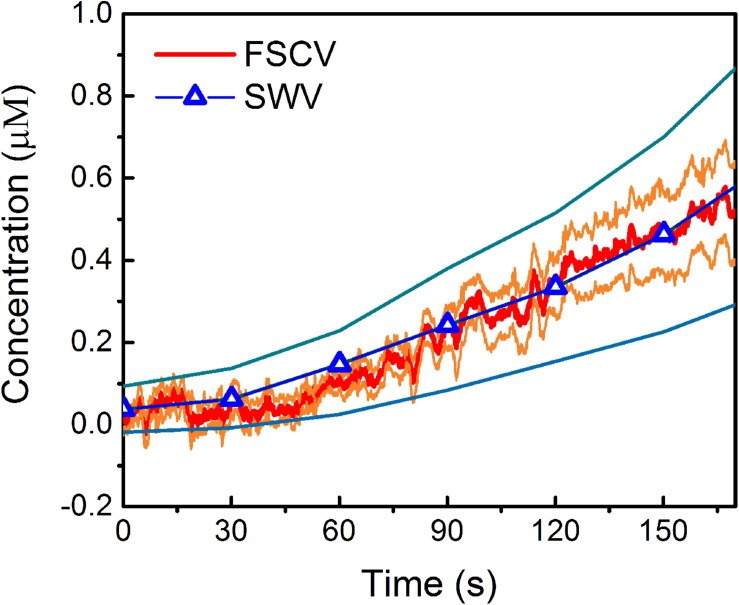
Comparison of *in vivo* MT detection using FSCV and SWV: Average *in vivo* MT concentration (±SEM), over time, detected at CFEs using FSCV (red) and SWV (blue) after the i.p. administration of 180 mg/kg MT. In these plots, *t* = 0 is the time of injection for both SWV and FSCV measurements. The orange and light-blue lines correspond to the min and max concentration trends, calculated as mean ± SEM (i.e., mean ± standard deviation divided by the square root of the sample size) for FSCV and SWV, respectively.

Remarkably, when comparing the *in vivo* MT concentrations detected using FSCV to those obtained in our previous SWV study (Castagnola et al., 2020), during the 170 s post i.p. injection of 180 mg/kg MT, we observed a very similar trend (Figure 5). The averaged detected concentrations at 170 s after MT administration were 0.55 μM (0.40 μM min, 0.64 μM max) for FSCV and 0.53 μM (0.32 μM min, 0.82 μM max) for SWV. It is worth noting that FSCV provides 240 times greater temporal resolution (0.1 versus 24 s) and 200 times higher sensitivity (28.1 versus 0.14 nA/μM) than SWV.

To provide a further validation of our electrochemical MT quantification, we compared the electrochemical results with microdialysis, by simultaneously performing microdialysis and FSCV experiments in the same location of same mice (*n* = 5). The dialysate, collected for 40 min before and after i.p. injection, was analyzed using LC-MS, i.e., injected onto an LC-MS and compared to a calibration curve. First of all, no MT peak was observed in baseline dialysate collected before the MT administration (Supplementary Figure 12). This is in agreement with the known low endogenous MT background in C57BL.6J mice (Roseboom et al., 1998). After MT injection, we obtained an average concentration of 4.3 μM (*n* = 5, 3.34 min and 6.69 max) from the dialysate collected for 40 min, which is on the same order of magnitude of the MT concentration detected with the SWV measurement [maximum detected concentration was 5.5 μM (4.4 μM min, 6.5 μM max) after 17.9 minutes following the MT administration] (Castagnola et al., 2020). It is worth pointing out that one cannot expect a perfect match between microdialysis and electrochemical results. Our electrochemical detections use pre-calibration, which may provide an underestimation of the MT *in vivo* concentration. On the other hand, the microdialysis result is an underestimation due to the impact of the microdialysis probes on the brain tissues and the corresponding recovery factor (Yang et al., 2000; Watson et al., 2006; Jaquins-Gerstl and Michael, 2015). Furthermore, there are clear differences in temporal resolution between the microdialysis (averaged over 40 min) and electrochemical methods (measured at subseconds for FSCV and seconds for SWV) (Watson et al., 2006; Jaquins-Gerstl and Michael, 2015; Puthongkham and Venton, 2020). Nevertheless, having the estimated MT concentrations in the same range supports the reliability of FSCV and SWV to measure MT in real time in mouse brain.

## Conclusion

In this study, we established a prolonged CFE electrochemical pre-conditioning protocol to enable stable FSCV background and demonstrated that pre-conditioning CFE with the Ross’s FSCV waveform overnight resulted in a stable sensitivity in response to 1 μM bolus injections of MT over a period of 8 h, or in the prolonged presence of a high MT concentration of 5 μM. Using the FSCV current and EIS as a predictor of the electrode/solution interface variations, we estimated the extent of fouling *in vivo*, and the effects of the tissue exposure on the impedance, capacitance, and sensitivity of the CFEs. We found no significant evidence of CFE fouling during the duration of the *in vivo* experiments, confirming the result obtained *in vitro*. Remarkably, *in vivo* measurements demonstrated a *drift-free* FSCV detection of exogenous MT in mouse brain for 3 min,004 more than twice the duration limit (usually < 90 s) reported in literature before FSCV background drifting occurs (Mark DeWaele et al., 2017; Meunier et al., 2019). The *in vivo* MT concentrations measured by FSCV are consistent with those measured by SWV upon the same MT injection regime (Castagnola et al., 2020), which is also supported by microdialysis. These results support the ability and reliability of FSCV to measure exogenously delivered MT *in real time* over several minutes *in vivo*.

## Data Availability Statement

The raw data supporting the conclusions of this article will be made available by the authors, without undue reservation.

## Ethics Statement

All procedures involving animals were approved by the Institutional Animal Care and Use Committee of the University of Pittsburgh.

## Author Contributions

EC led *in vitro* and *in vivo* experiments, performed *in vitro* and *in vivo* fouling experiments and data analysis, and wrote the manuscript. ER fabricated microdialysis probes and led microdialysis experiments and analysis. KW performed parts of the surgeries and SEM imaging. AG performed parts of the surgeries. MM performed impedance measurements and *in vitro* calibrations and helped with CFEs fabrication. IT fabricated the CFEs, supervised the *in vitro* and fouling experiments, and edited the manuscript. AM supervised the microdialysis experiments and edited the manuscript. XTC supervised the project, guided the data analysis, and edited the manuscript. All authors contributed to the article and approved the submitted version.

## Conflict of Interest

The authors declare that the research was conducted in the absence of any commercial or financial relationships that could be construed as a potential conflict of interest.
