# Supplementary material for: Real-Time Fast Scan Cyclic Voltammetry Detection and Quantification of Exogenously Administered Melatonin in Mice Brain
Source: Front Bioeng Biotechnol. 2020 Nov 24;8:602216. doi: 10.3389/fbioe.2020.602216 (PMC7732424; doi:10.3389/fbioe.2020.602216)
Supplement: Supplementary file 1 [file Data_Sheet_1.PDF]

Supplementary Information

Real-time Fast Scan Cyclic Voltammetry detection and quantification of exogenously administered melatonin in mice brain

Elisa Castagnola[1], Elaine M. Robbins[2], Kevin Woeppel[1,3], Moriah McGuier[4], Asiyeh Golabchi[1,3], I. Mitch Taylor[4], Adrian C. Michael[2], X. Tracy Cui[1,3,5*]

[1] Department of Bioengineering, University of Pittsburgh, Pittsburgh, PA 15260, USA

[2] Department of Chemistry, University of Pittsburgh, Pittsburgh, PA 15260, USA

[3] Center for Neural Basis of Cognition, University of Pittsburgh, Pittsburgh, PA 15213, USA

[4] Department of Chemistry, Saint Vincent College, Latrobe, PA 15650, USA

[5] McGowan Institute for Regenerative Medicine, University of Pittsburgh, Pittsburgh, PA 15219, USA

*** Correspondence:**

X. Tracy Cui

xic11@pitt.edu

Phone: (412) 383-6672

Keywords: *Fast Scan Cyclic Voltammetry, Melatonin, Fouling, Carbon Fiber Microelectrodes, Brain, Electrochemical Impedance Spectroscopy*

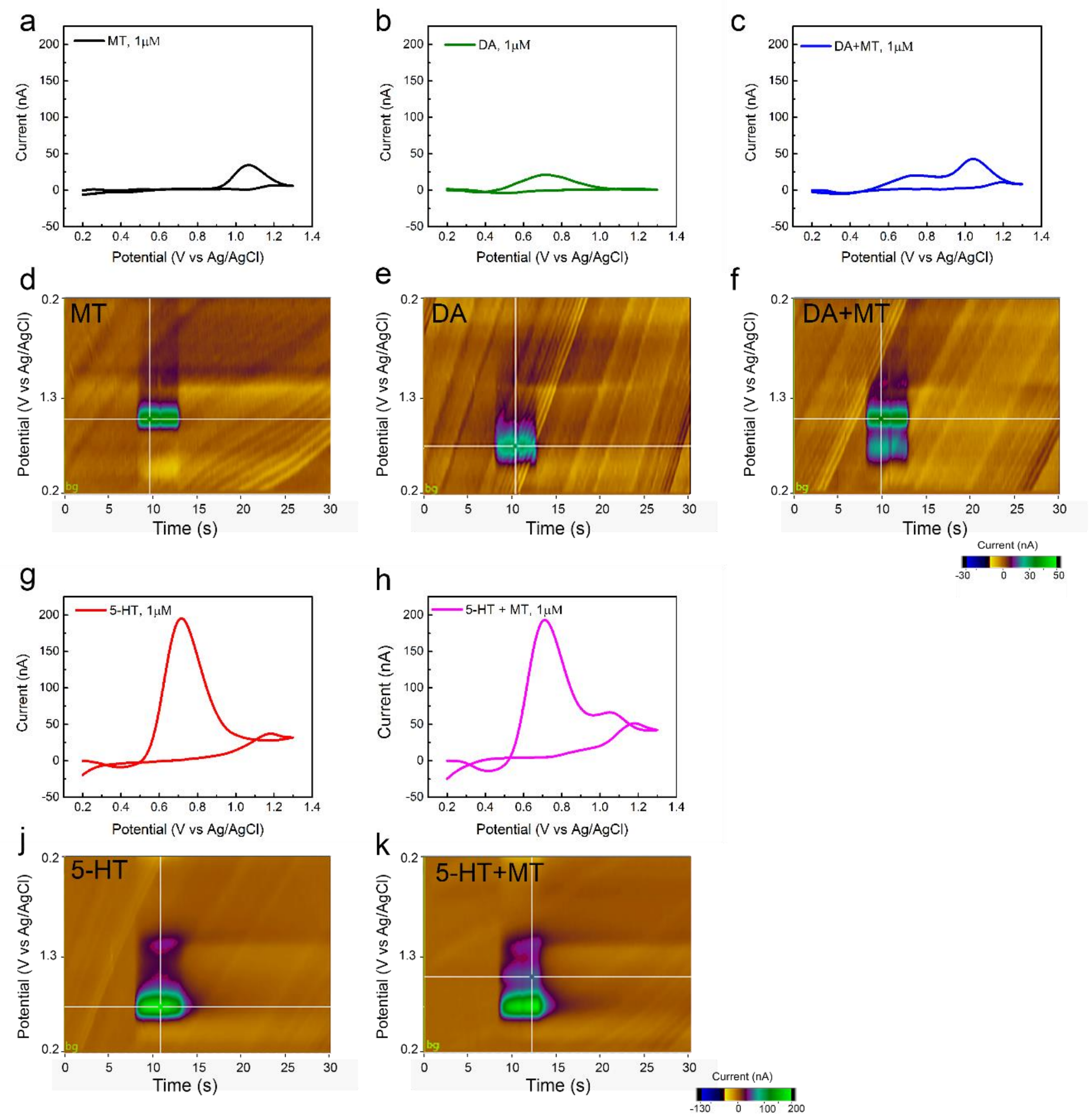

Supplementary Figure 1. MT fast scan cyclic voltammetry (FSCV) selectivity: MT can be distinguished among the most common electroactive neurotransmitter in the brain, i.e. dopamine (DA) and serotonin (5-HT). Background subtracted CVs and the respective color plots for 1µM bolus injection of MT (a, d), DA (b, e), 5-HT (c, f) and their relative mixtures (c, f, i, k) show that MT can be discriminate from DA and 5-HT. DA presents an oxidation potential peak around 0.6V and is poorly detected using this waveform. 5-HT presents its oxidation potential peak at 0.7 V, well separated from the MT peak, but it presents higher sensitivity at CFE respect MT.

1. *MT FSCV Selectivity*

We first ruled out ascorbic acid (AA) and uric acid (UA) as interference agents in the FSCV experiments, because they are considered nonadsorbing species with slow reaction kinetics, resulting in the faradaic signal being lost in the background at the very high scan rate of FSCV (Bath et al., 2000; Robinson et al., 2003; Huffman and Venton, 2008). Furthermore, their concentration in the brain, potentially 100-1000 times higher than other neurotransmitters, are not supposed to present fast variations, and are thus subtracted by sub-second resolution techniques that can read only sub-second changes in concentration (Bath et al., 2000; Robinson et al., 2003; Huffman and Venton, 2008).

DA and 5-HT are present in different areas of the brain and they are related to several disease states, such as Parkinson's disease, depression and addiction disorders (Politis and Loane, 2011; Koob and Volkow, 2016; Belujon and Grace, 2017). Background subtracted CVs and the respective color plots for 1μM bolus injection of MT, DA, 5-HT and their relative mixtures are reported in Supplementary Figure 2a-k.

As previous discussed by A. L. Hensley et al. (Austin L., 2018), DA and MT can be easily discriminated. DA presents an oxidation potential peak around 0.6V and is poorly detected using this waveform because it requires a negative holding potential in order to optimize DA adsorption at CFEs.

Tonic DA concentrations have been estimated to be 82 ± 6 nM (using square wave voltammetry performed at PEDOT/CNT-functionalized CFEs) in the rat dorsal striatum (DS) (Taylor et al., 2019), 90 ± 9 nM (Atcherley et al., 2015) and 41 ± 13 nM (Johnson et al., 2018) in the nucleus accumbens (NAc) of mice and rats, using fast scan controlled adsorption voltammetry (FSCAV) and convolution-based FSCV, respectively. Considering that the striatum is one of the brain regions with higher DA concentrations and that the Ross FSCV waveform is not optimized for DA detection, these concentration ranges (41-207 nM), should not significantly affect the MT current. However, when electrically evoked, by stimulating the ipsilateral MFB, DA has been detected both in the NAc (Hashemi et al., 2012) and DS (Mitch Taylor et al., 2012) of rats in high (3-4 μM) concentrations.

However, when electrically evoked, by stimulating the ipsilateral MFB, DA has been detected both in the NAc (Hashemi et al., 2012) and dorsal striatum (Mitch Taylor et al., 2012) of rats in high 3-4 μM concentrations.

For this reason, to access the interfering effects from such high concentration of DA, we tested a mixture of 0.5uM of MT (detected in this study) and 4μM DA (to simulate a high DA concentration evoked by electrical stimulation). The results are reported in Supplementary Figure 2 a, c and demonstrated that it is possible to discriminate 0.5 μM MT in presence of 4 μM DA concentration.

5-HT, the MT precursor, presents its oxidation potential peak at 0.7 V, which is well separated from the MT peak. However, CFEs have shown high sensitivity to 5-HT using this FSCV waveform and high 5-HT concentration may still cause an overlapping background in oxidation current.

Basal 5-HT levels in the CA2 region of the hippocampus have been estimated as 64.9 ± 2.3 nM in mice (Abdalla et al., 2017). Electrical stimulation of the MFB has been shown to evoke 5-HT at ~300 times smaller concentrations than that of DA (Hashemi et al., 2012) (10-20 nM), in the ipsilateral SNr. Thus, we decided to test a mixture of 0.5 μM of MT (detected in this study) and 0.1 μM of 5-HT, higher than what is observed for tonic and phasic 5-HT concentrations in the brain. The results are reported in Supplementary Figure 2 b, d and show that it is possible to discriminate MT also in presence of 5-HT concentration similar to what present in physiological conditions.

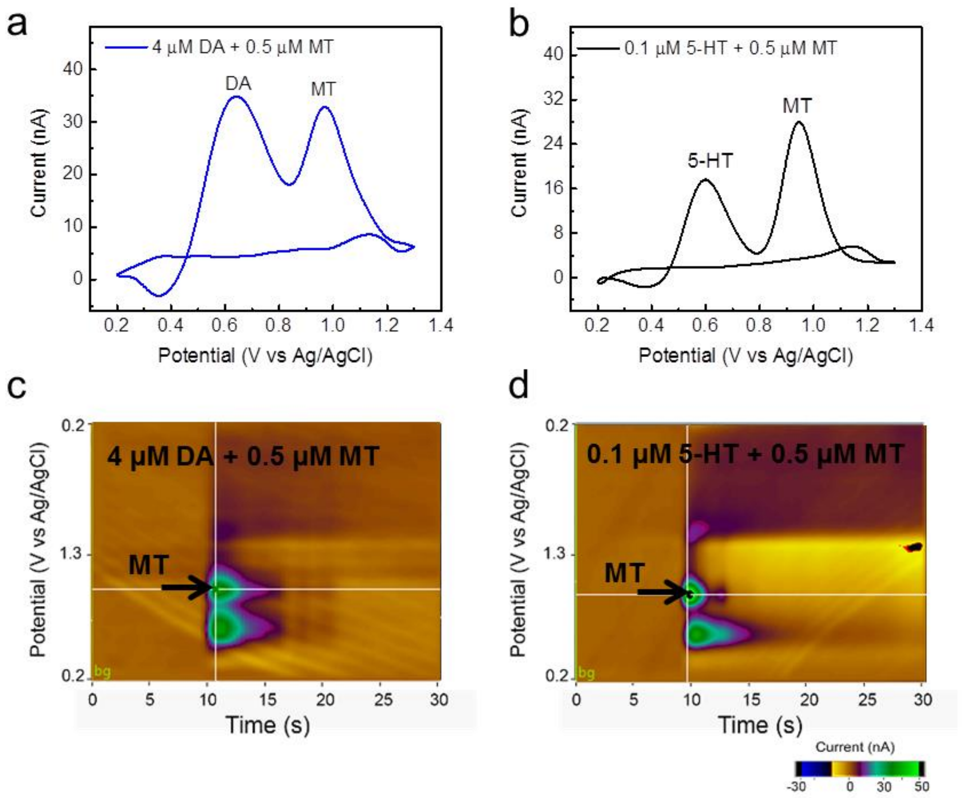

Supplementary Figure 2. MT fast scan cyclic voltammetry (FSCV) selectivity: MT can be distinguished among the most common electroactive neurotransmitter in the brain, i.e. dopamine (DA) and serotonin (5-HT). Background subtracted CVs and the respective color plots for a mixture of 0.5µM MT and 4 µM DA (a, c), and for a mixture of 0.5µM MT and 0.1 µM 5-HT (b, d) show that MT can be discriminate from DA and 5-HT physiological concentrations.

Norepinephrine (NE), and epinephrine (EP) are catecholamines that generate similar voltammetric features of DA, using FSCV triangular waveform at CFEs, which should not overlap with the MT peak at 1.03 V (Roberts and Sombers, 2018). Similarly, the precursors and metabolites for these catecholamines, including 3,4-dihydroxyphenylacetic acid (DOPAC) and homovanillic acid (HVA) have similar structures and generate similar voltammograms, around 0.6 V, when detected using a simple triangular waveform (Roberts and Sombers, 2018). Furthermore, they are not present in high concentrations in the brain (Roberts and Sombers, 2018) and are currently only detectable with microdialysis coupled with liquid chromatography with relatively low temporal and spatial resolution (Fang et al., 2013).

Additionally, hydrogen peroxide (H_2O_2) fluctuations, indicating the presence of tissue dysfunctions (Spanos et al., 2013), may potentially affect the determination of MT in the brain. However, physiologically relevant H_2O_2 concentrations measured in the extracellular space of the brain are in the range of a few µM (Kulagina and Michael, 2003), similar or lower than the detection limit of CFEs (Sanford et al., 2010) and carbon microdisc electrodes (Roberts et al., 2011). Furthermore, the H_2O_2 oxidation peak is at 1.2V during FSCV measurements with a required switching potential limit greater than 1.2 V to sufficiently oxidize the CFE surface for the detection of H_2O_2 (Sanford et al., 2010). For these reasons, FSCV measurements of H_2O_2 *in vivo*, in the striatum of rat, has been measured only after pharmacological manipulation or micro-infusion (Spanos et al., 2013), and are unlikely to interfere with MT detection in physiological conditions. We also considered the possibility of discriminating MT in presence of the MT metabolites in the brain that can be produced due to the administration of melatonin.

The metabolism of MT in the central nervous system follows several pathways, with the consequent formation of different metabolites (Hardeland, 2010). For example, it has been demonstrated that melatonin can be demethylated to N-acetylserotonin (NA-5HT, Molecular Weight 218.25 g/mol), or produce 6-hydroxymelatonin (6-HMA, Molecular Weight 248.28 g/mol). 6-HMA oxidizes at the same potential as MT, however, the Ross' group demonstrated that, using this specific waveform, CFEs are 6-fold more sensitive to MT than to 6-HMA. Therefore, the presence of 6-HMA will not significantly impact the melatonin signal (Austin L., 2018). Furthermore, an earlier study did not observe 6-HMA among the products of administered MT (Hirata et al., 1974), while another study found very low levels of 6-HMA in the cerebral cortex of mice (0.1% of corresponding MT) (Lahiri et al., 2004). Thus, it is unlikely that 6-HMA will compromise the MT detection. On the other hand, NA-5HT is structurally similar to MT, but oxidizes at 0.7 V, and can be clearly separated from MT when melatonin is in 5-fold excess, by using this FSCV waveform (Austin L., 2018).

MT deacetylation to 5-MT has been observed in certain areas of the CNS, and it seems to exist also in the pineal gland of various vertebrates; however, this pathway plays only a minor role (Hardeland, 2010).

Among the vertebrate organs studied, a relevant rate of melatonin deacetylation was only observed in the retinas of fish, amphibians and reptiles (Hardeland, 2010).

MT can also undergo non-enzymatic hydroxylation and nitrosation by interacting with reactive oxygen and nitrogen species (RONS). One major RONS that reacts with MT is hydroxyl radicals (Tan et al., 2007; Hardeland, 2010). Among the reported products of the reaction between MT and hydroxyl radicals, cyclic 3-hydroxymelatonin (3-OHM)(Tan et al., 1999; Reiter et al., 2001; Hardeland, 2010; Galano et al., 2014) and 2-Oxomelatonin (or N-[2-(2-Oxo-5-methoxyindoline-3-yl)ethyl]acetamide) (Fourtillan, 2002; Hardeland, 2010) have been detected in the urine of rat and mice after administration of exogenous melatonin.(Tan et al., 1999; Ma et al., 2008; Hardeland, 2010).Their molecular weights are 248.28g/mol and 276.29 g/mol respectively, sufficiently different from MT (232.28g/mol). We did not observe molecules corresponding to these molecular weights in the dialysate, nor did we observe species with molecular weights of 6-HMA and NA-5-HT, suggesting negligible presence of these metabolites.

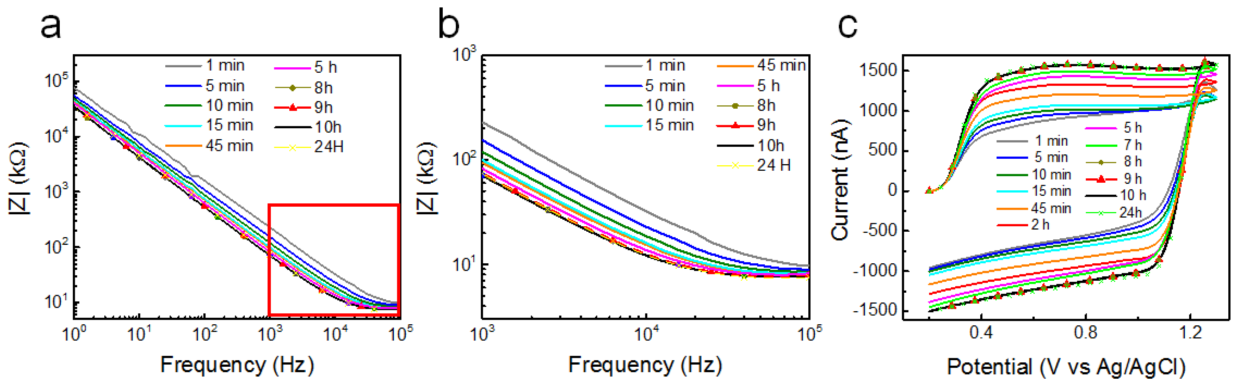

Supplementary Figure 3. Effect of the electrochemical pre-conditioning on CFE impedance and non-subtracted FSCV. a) Plot of the impedance spectra (magnitude of impedance versus frequency) of CFE during the prolonged CFE pre-conditioning at different time points, i.e. 1, 5, 10, 15, 45 minutes, 5, 8, 9, 10 and 24 hours of FSCV cycling. During the prolonged CFE pre-conditioning, the FSCV waveform applied is the Ross' waveform, i.e. scanning the potential from

0.2 to 1.3 V versus Ag/AgCl and back at 600 V/s (10 Hz). b) magnification of the impedance spectra, corresponding to the red square in a). c) Corresponding non-subtracted FSCV during the prolonged CFE pre-conditioning at different time points, i.e. 1, 5, 10, 15, 45 minutes, 2, 5, 7, 8, 9, 10 and 24 hours of FSCV cycling.

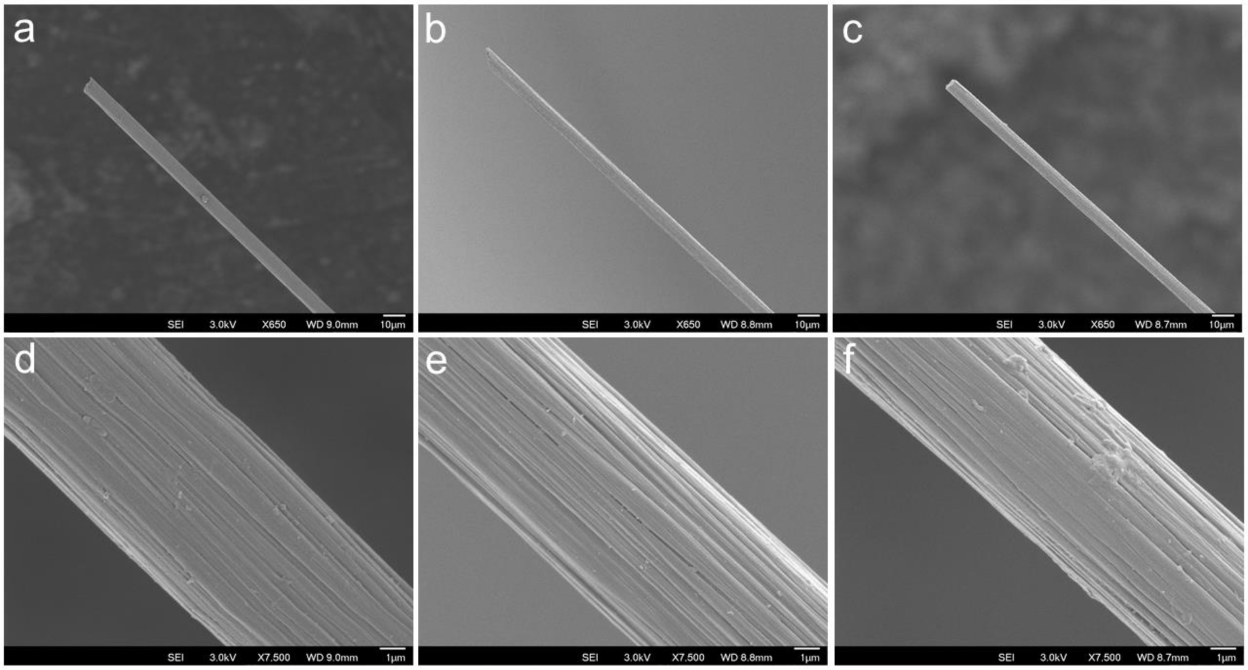

Supplementary Figure 4. **Effect of the electrochemical pre-conditioning on CFE surface.** SEM images of a, d) pristine CFE, b, e) a representative CFE at which the Ross' waveform was applied overnight (≥ 12 hr) at 600 V/s, and c, f) a representative CFE at which a more aggressive FSCV waveform (-0.5 to 1.9 V versus Ag/AgCl and back at 400 V/s) was applied for two minutes. The integrity of the CFE seems to be preserved after both the electrochemical treatments.

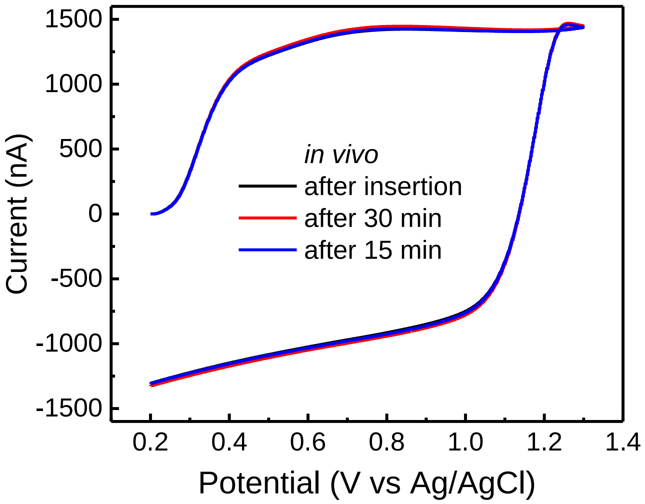

Supplementary Figure 5. FSCV *in vivo*: non-subtracted background CV of CFEs (average, n=5) 1 minute after implant and impedance measurement in the brain and after 15 and 30 minutes of FSCV *in vivo*, respectively (before MT administration).

2. *Electrochemical Impedance Spectroscopy (EIS): non-ideal Randles model*

The best fit for the collected EIS data was obtained using a *non-ideal* Randles. The modified Randles circuit is composed of uncompensated electrode - solution resistance (Rsol+carbon) in series with a parallel combination of charge-transfer resistance (Rct), constant phase element (ZCPE), and Warburg impedance (Zw) (MacDonald, 1987; Nimbalkar et al., 2018; Meunier et al., 2020). However, here the system impedance is dominated by the double layer capacitance (ZCPE) and we do not observe diffusional processes at low frequencies, that should be described by a Warburg element. Thus, Rct and Zw terms are omitted. This non-ideal Randles equivalent circuit (Supplementary Figure 3 inset) is consistent with results reported in the literature for CFEs glue-sealed at the tip (Meunier et al., 2020).

The constant phase element Z_{CPE} is define as: $Z_{CPE} = 1/(j\omega)^n\ Y_o$, where Y_o represents the double-layer capacitance and n is a constant related to the angle of rotation in the complex plane compared to the purely capacitive behavior (n = 1 for a pure capacitor) (MacDonald, 1987; Nimbalkar et al., 2018; Meunier et al., 2020). This circuit element exists mostly to describe capacitance as it appears in real electrochemical systems, an "imperfect capacitance", because of rough surfaces, presence of defect or a distribution of reaction rates (Meunier et al., 2020).

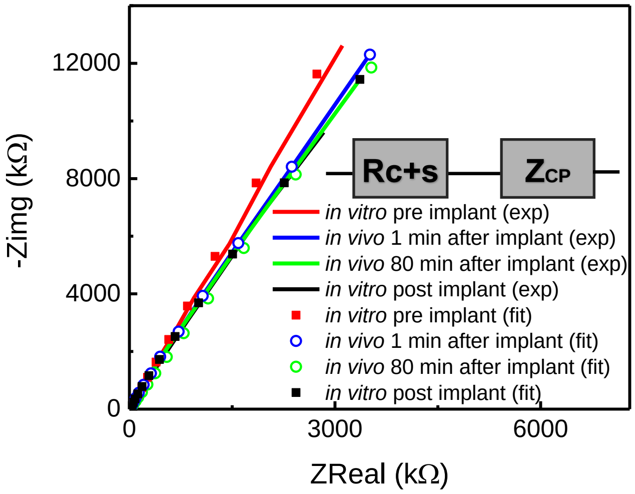

Supplementary Figure 6. Average Nyquist plot (n=6, 5 Hz - 100 kHz) of CFEs measured before implant in vitro in aCSF (red), 1 minute after the implant in vivo (blue), at the end of the experimental session (≥

80 minutes after implant) in vivo (green), and in vitro after the extraction from the brain (post implant, black). The experimental data are reported in continuous lines and the corresponding curve-fitting in scattered points. The equivalent circuit model is reported in inset.

The EIS measurements of CFEs (in aCSF) post implant revealed that $R_{sol+carbon}$ and n values were not totally restored after the extraction from the brain (Supplementary Figure 3, Table 1), with a significant impedance variation respect the pre-implant EIS (significant, two-way ANOVA with repeated measures: $F(1, 156) = 62.15$, $P<0.0001<0.05$), similar to what was previously reported by the Sombers' group (Meunier et al., 2020). Accordingly, the post implant FSCV capacitance showed a 11.6% decrease (Supplementary Figure 4 and 6 c) and a significant decrease in sensitivity (Supplementary Figure 6), similarly to our previous observations (Castagnola et al., 2020a). Upon electrochemical cleaning of the CFEs stripping off the pulled-out tissue (Castagnola et al., 2020b; Meunier et al., 2020), the CFEs impedance and sensitivity can be electrochemically restored (Supplementary Figure 6). More details about the electrochemical cleaning/restoration and how it can affect the CFE's sensitivity to MT has been reported in the Supplementary Information (Supplementary Figure 6 and 7 and Section 3). In summary, we tested whether an electrochemical cleaning procedure can be used to regenerate the CFE surface, by removing the biological encapsulation, and we can conclude that it is effective without permanently modifying the CFE sensitivity.

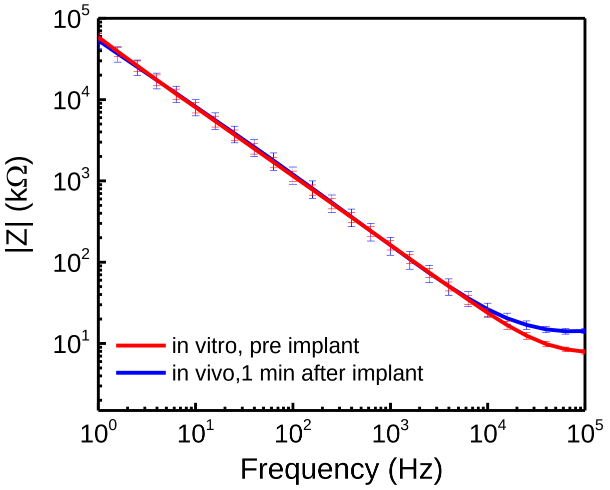

Supplementary Figure 7 Effect of the tissue exposure on the solution/CFE interface resistance. Plot of the impedance spectra (magnitude impedance versus frequency) of CFEs (n=6, 1Hz - 100 kHz) measured in vitro before implant in aCSF (red), and 1 minute after implant *in vivo* in the brain visual cortex (blue).

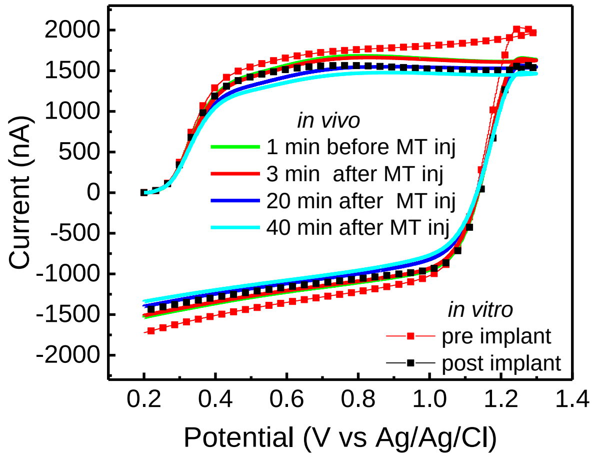

Supplementary Figure 8. Representative example of a not-background subtracted FSCV in aCSF in vitro, before (red, scattered points) and post (black, scattered points) implant in the brain, and in vivo, at different time points: immediately before MT injection (green), and after 3 (red), 20 (blue) and 40 (cyan) minutes following MT administration.

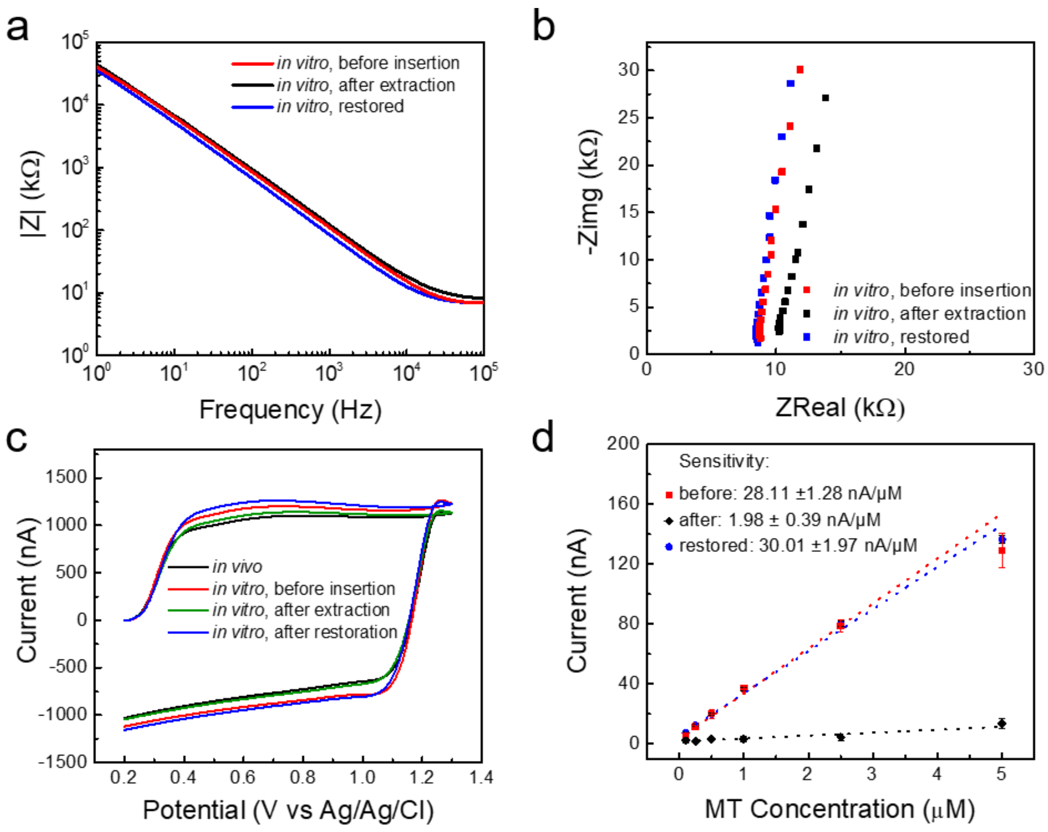

Supplementary Figure 9. CFEs electrochemical restoration: a, b) A representative example of the impedance magnitude (a) and Nyquist plot (b) of the same CFE **1.** before insertion into the brain (in aCSF), **2.** after the explant from the brain, in aCSF, and 3. after electrochemical cleaning/restoration. **c)** the corresponding FSCV background current, in comparison with the background current recorded *in vivo*. **d)** *in vitro* FSCV MT calibration curves conducted at CFEs before (28.11 ± 1.28 nA/μM), after the *in vivo* experiments (1.98 ± 0.39 nA/μM) and after the electrochemical cleaning (30.01 ± 1.97 nA/μM). Before insertion and after restoration, the average (n=5) sensitivity (background subtracted peak current vs MT concentration) is linearly correlated. After extraction from the brain, the sensitivity drops 93% and it is not linear. The electrochemical cleaning/restoration has been performed with the aim to remove the biological encapsulation after the CFE extraction from the brain. The electrochemical cleaning procedure consists of applying a FSCV waveform scanning the potential from -0.5 to 1.9 V versus Ag/AgCl and back at 400 V/s for two minutes or apply the Ross Waveform overnight at 600V/s.

3. The electrochemical cleaning (restoration) procedure

This procedure consists of applying apply the Ross' waveform overnight (≥12h) at 600V/s or, more aggressively, a FSCV waveform scanning the potential from -0.5 to 1.9 V versus Ag/AgCl and back at 400 V/s for two minutes, in order to renew the CFE surface.

We tested whether the aggressive cleaning procedure permanently affects the CFE's sensitivity to MT. The sensitivity of pristine CFEs to 1 µM MT has been measured before and after (at different time points) the electrochemical cleaning procedure, consisting of applying a FSCV waveform scanning the potential from -0.5 to 1.9 V versus Ag/AgCl and back at 400 V/s for two minutes. Immediately after the procedure, we observed a 3-fold increase in sensitivity, that is restored to the original value after 2h (Supplementary Figure 5). Thus, we can conclude that the electrochemical cleaning can be used to regenerate the surface and remove the biological encapsulation, without permanently modifying the CFE sensitivity. We would like to note that the calibration reported in Supplementary Figure 4a has been measured several hours after the electrochemical restoration.

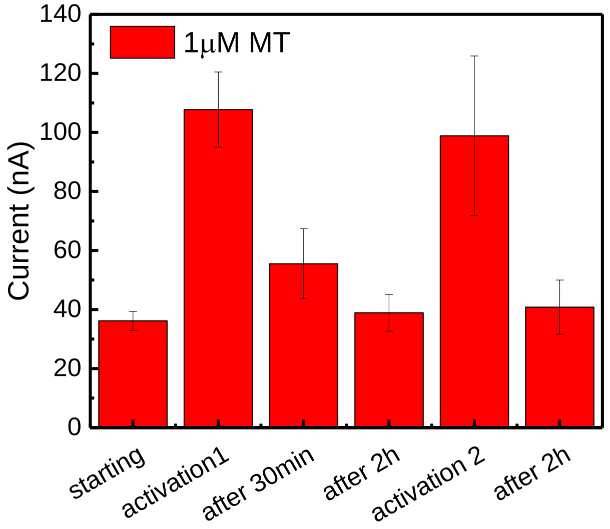

Supplementary Figure 10. Reversibility of the CFEs electrochemical cleaning: the sensitivity to 1μM MT of pristine CFEs has been tested before and after (different time points) the electrochemical cleaning procedure, consisting of applying a FSCV waveform scanning the potential from -0.5 to 1.9 V versus Ag/AgCl and back at 400 V/s for two minutes. The electrochemical cleaning can be used to regenerate the surface and remove the biological encapsulation, without permanently modified the CFE sensitivity.

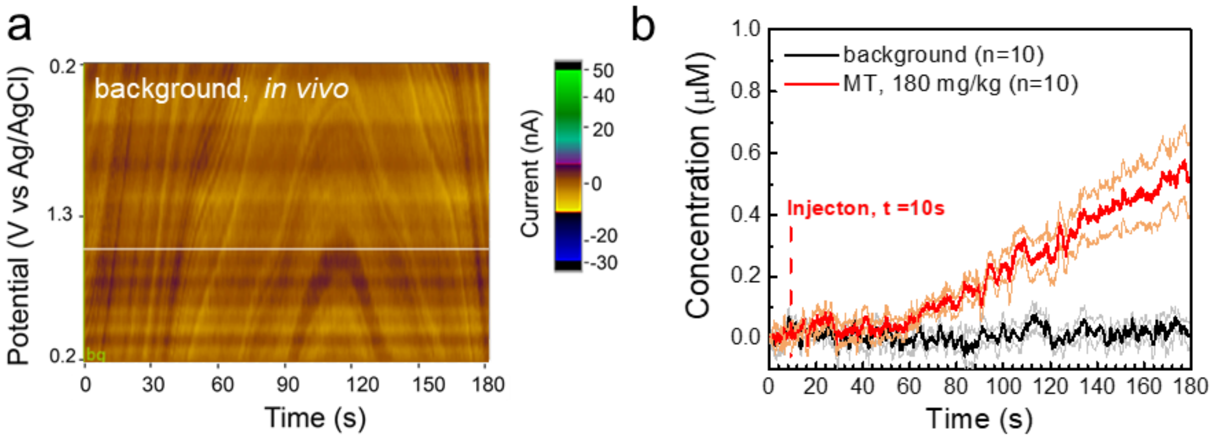

Supplementary Figure 11. **In vivo FSCV current background stability: a**) representative color plot for a 3-minute FSCV current background collection in vivo shows a stable background without observable current drift. b) Average in vivo MT concentration (±SEM), over time, detected at CFEs using FSCV before and after the i.p. administration of 180 mg/kg MT (red, n=10) at t=10 s, in comparison with the average FSCV background in vivo (black, n=10), collected on the same animals before the MT detection experiment. CFEs started to detect an increase in MT concentration about 50 seconds following the i.p. injection and reach the concentration of 0.51 μM (0.40 μM min, 0.62 μM max) 170 s following MT administration.

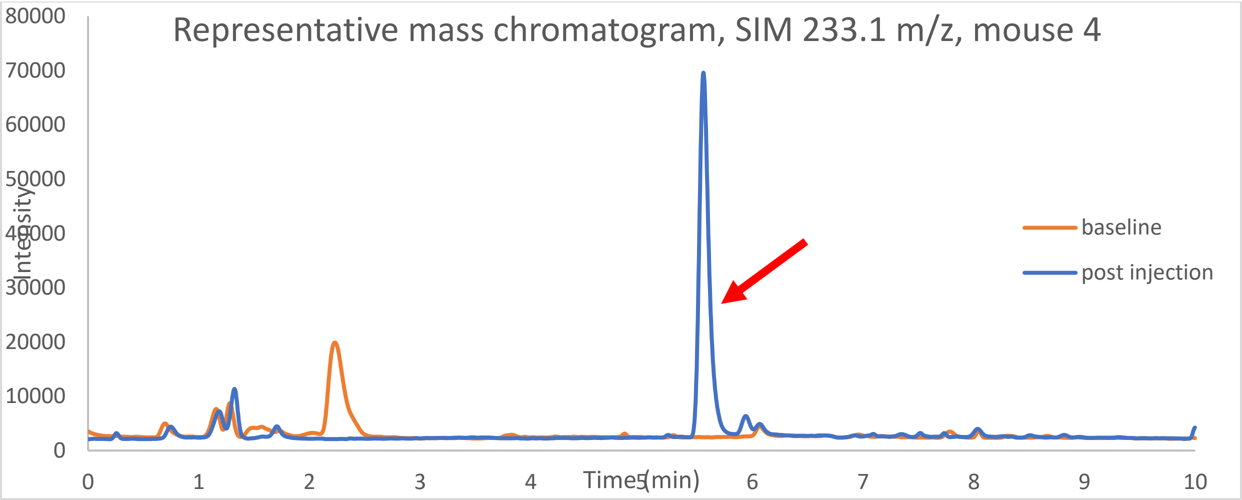

Supplementary Figure 12: Representative mass chromatogram shows that there is no MT peak (red arrow) in the baseline.

Atcherley, C.W., Wood, K.M., Parent, K.L., Hashemi, P., and Heien, M.L. (2015). The coaction of tonic and phasic dopamine dynamics. *Chemical Communications* 51(12), 2235-2238.

Austin L., H., Adam R. Colley, and Ashley E. Ross (2018). Real-Time Detection of Melatonin Using Fast-Scan Cyclic Voltammetry. *Anal. Chem.* 90, 8642−8650.

Bath, B.D., Michael, D.J., Trafton, B.J., Joseph, J.D., Runnels, P.L., and Wightman, R.M. (2000). Subsecond adsorption and desorption of dopamine at carbon-fiber microelectrodes. *Analytical chemistry* 72(24), 5994-6002.

Belujon, P., and Grace, A.A. (2017). Dopamine system dysregulation in major depressive disorders. *International Journal of Neuropsychopharmacology* 20(12), 1036-1046.

Castagnola, E., Woeppel, K., Golabchi, A., McGuier, M., Chodapaneedi, N., Metro, J., et al. (2020a). Electrochemical detection of exogenously administered melatonin in the brain. *Analyst* 145(7), 2612-2620.

Castagnola, E., Woeppel, K., Golabchi, A., McGuier, M., Chodapaneedi, N., Metro, J., et al. (2020b). Electrochemical detection of exogenously administered melatonin in the brain. *Analyst*.

Fang, H., Pajski, M.L., Ross, A.E., and Venton, B.J. (2013). Quantitation of dopamine, serotonin and adenosine content in a tissue punch from a brain slice using capillary electrophoresis with fast-scan cyclic voltammetry detection. *Analytical Methods* 5(11), 2704-2711.

Fourtillan, J.B. (2002). Role of melatonin in the induction and maintenance of sleep. *Dialogues in Clinical Neuroscience* 4(4), 395.

Galano, A., Tan, D.X., and Reiter, R.J. (2014). Cyclic 3-hydroxymelatonin, a key metabolite enhancing the peroxyl radical scavenging activity of melatonin. *RSC advances* 4(10), 5220-5227.

Hardeland, R. (2010). Melatonin metabolism in the central nervous system. *Current Neuropharmacology* 8(3), 168-181.

Hashemi, P., Dankoski, E.C., Lama, R., Wood, K.M., Takmakov, P., and Wightman, R.M. (2012). Brain dopamine and serotonin differ in regulation and its consequences. *Proceedings of the National Academy of Sciences* 109(29), 11510-11515.

Hirata, F., Hayaishi, O., Tokuyama, T., and Senoh, S. (1974). In vitro and in vivo formation of two new metabolites of melatonin. *Journal of Biological Chemistry* 249(4), 1311-1313.

Huffman, M.L., and Venton, B.J. (2008). Electrochemical Properties of Different Carbon-Fiber Microelectrodes Using Fast-Scan Cyclic Voltammetry. *Electroanalysis: An International Journal Devoted to Fundamental and Practical Aspects of Electroanalysis* 20(22), 2422-2428.

Johnson, J.A., Rodeberg, N.T., and Wightman, R.M. (2018). Measurement of basal neurotransmitter levels using convolution-based nonfaradaic current removal. *Analytical chemistry* 90(12), 7181-7189.

Koob, G.F., and Volkow, N.D. (2016). Neurobiology of addiction: a neurocircuitry analysis. *The Lancet Psychiatry* 3(8), 760-773.

Kulagina, N.V., and Michael, A.C. (2003). Monitoring hydrogen peroxide in the extracellular space of the brain with amperometric microsensors. *Analytical chemistry* 75(18), 4875-4881.

Lahiri, D.K., Ge, Y.W., Sharman, E.H., and Bondy, S.C. (2004). Age-related changes in serum melatonin in mice: higher levels of combined melatonin and 6-hydroxymelatonin sulfate in the cerebral cortex than serum, heart, liver and kidney tissues. *Journal of pineal research* 36(4), 217-223.

Ma, X., Chen, C., Krausz, K.W., Idle, J.R., and Gonzalez, F.J. (2008). A metabolomic perspective of melatonin metabolism in the mouse. *Endocrinology* 149(4), 1869-1879.

MacDonald, J.R. (1987). Impedence Spectroscopy--Emphasizing Solid Materials and Systems. *Wiley-Interscience, John Wiley and Sons*, 1-346.

Meunier, C.J., Denison, J.D., McCarty, G.S., and Sombers, L.A. (2020). Interpreting Dynamic Interfacial Changes at Carbon Fiber Microelectrodes Using Electrochemical Impedance Spectroscopy. *Langmuir*.

Mitch Taylor, I., Jaquins-Gerstl, A., Sesack, S.R., and Michael, A.C. (2012). Domain-dependent effects of DAT inhibition in the rat dorsal striatum. *Journal of neurochemistry* 122(2), 283-294.

Nimbalkar, S., Castagnola, E., Balasubramani, A., Scarpellini, A., Samejima, S., Khorasani, A., et al. (2018). Ultra-capacitive carbon neural probe allows simultaneous long-term electrical stimulations and high-resolution neurotransmitter detection. *Scientific reports* 8(1), 1-14.

Politis, M., and Loane, C. (2011). Serotonergic dysfunction in Parkinson's disease and its relevance to disability. *The Scientific World Journal* 11, 1726-1734.

Reiter, R.J., Tan, D.-x., Manchester, L.C., and Qi, W. (2001). REVIEW ARTICLES-Biochemical Reactivity of Melatonin with Reactive Oxygen and Nitrogen Species: A Review of the Evidence. *Cell Biochemistry and Biophysics* 34(2), 237-256.

Roberts, J.G., Hamilton, K.L., and Sombers, L.A. (2011). Comparison of electrode materials for the detection of rapid hydrogen peroxide fluctuations using background-subtracted fast scan cyclic voltammetry. *Analyst* 136(17), 3550-3556.

Roberts, J.G., and Sombers, L.A. (2018). Fast-scan cyclic voltammetry: chemical sensing in the brain and beyond. *Analytical chemistry* 90(1), 490-504.

Robinson, D.L., Venton, B.J., Heien, M.L., and Wightman, R.M. (2003). Detecting subsecond dopamine release with fast-scan cyclic voltammetry in vivo. *Clinical chemistry* 49(10), 1763-1773.

Sanford, A.L., Morton, S.W., Whitehouse, K.L., Oara, H.M., Lugo-Morales, L.Z., Roberts, J.G., et al. (2010). Voltammetric detection of hydrogen peroxide at carbon fiber microelectrodes. *Analytical chemistry* 82(12), 5205-5210.

Spanos, M., Gras-Najjar, J., Letchworth, J.M., Sanford, A.L., Toups, J.V., and Sombers, L.A. (2013). Quantitation of hydrogen peroxide fluctuations and their modulation of dopamine dynamics in the rat dorsal striatum using fast-scan cyclic voltammetry. *ACS chemical neuroscience* 4(5), 782-789.

Tan, D.-X., Manchester, L., Reiter, R., and Plummer, B. (1999). Cyclic 3-hydroxymelatonin: a melatonin metabolite generated as a result of hydroxyl radical scavenging. *Neurosignals* 8(1-2), 70-74.

Tan, D.X., Manchester, L.C., Terron, M.P., Flores, L.J., and Reiter, R.J. (2007). One molecule, many derivatives: a never-ending interaction of melatonin with reactive oxygen and nitrogen species? *Journal of pineal research* 42(1), 28-42.

Taylor, I.M., Patel, N.A., Freedman, N.C., Castagnola, E., and Cui, X.T. (2019). Direct in vivo electrochemical detection of resting dopamine using Poly (3, 4-ethylenedioxythiophene)/Carbon Nanotube functionalized microelectrodes. *Analytical chemistry* 91(20), 12917-12927.
